# Evaluation of Antioxidant, Anti-inflammatory, and Antimicrobial Activities of Raspberry Fruit Extract: An In Vitro Study

**DOI:** 10.7759/cureus.54045

**Published:** 2024-02-12

**Authors:** R. Gomathi, T N Umamaheswari, Roland Prethipa

**Affiliations:** 1 Department of Oral Medicine and Radiology, Saveetha Dental College and Hospitals, Saveetha Institute of Medical and Technical Sciences (SIMATS), Chennai, IND

**Keywords:** health, plants, biomedical applications, antioxidant, antimicrobial, raspberry

## Abstract

Introduction

*Rubus idaeus (R. idaeus)*is an edible fruit that contains numerous significant bioactive compounds that hold important biological properties and are categorized as nutraceuticals owing to the health benefits it imparts including decreasing the risk of chronic diseases like diabetes mellitus, cancer, heart disease, and many other diseases. The objective of the present research was to explore the antimicrobial, antioxidant, and anti-inflammatory characteristics of the aqueous raspberry extract through in vitro assays.

Materials and methods

*R. idaeus* aqueous extract was prepared and examined for its antimicrobial activity against *Streptococcus mutans (S. mutans)* bacteria and *Candida albicans (C. albicans)* fungi using the agar-well diffusion method, and the antioxidant activity was evaluated using the DPPH (2, 2-Diphenyl-1-picrylhydrazyl hydrate) radical scavenging assay and the hydrogen peroxide radical scavenging assay. The anti-inflammatory activity of the prepared extract was investigated using bovine serum albumin (BSA) and egg albumin denaturation assays.

Results

*R. idaeus* fruit extract displayed strong antimicrobial activity at a higher concentration of 100 µL with a 26 mm zone of inhibition against *Streptococcus mutans* and 24 mm for* Candida albicans.* The extract showed 87.42% hydrogen peroxide free radical scavenging activity and inhibited 91.12% DPPH free radicals at the highest concentration of 50 µg/mL. The extract showed effective anti-inflammatory activity by preventing the denaturation of bovine serum albumin (80%) and egg albumin proteins (77%) at the highest concentration of 50 μg/mL. The free radical scavenging activity positively correlates with the increased concentration of the prepared extract against DPPH and hydrogen peroxide free radicals, thus showing the raspberry extract's potent antioxidant activity. Similarly, the anti-inflammatory assay result shows that the prepared raspberry aqueous extract has excellent anti-inflammatory activity by preventing the denaturation of bovine serum albumin and egg albumin protein in a dose-dependent manner.

Conclusion

The meticulously prepared raspberry extract exhibited noteworthy antimicrobial, antioxidant, and anti-inflammatory characteristics, and owing to its astounding therapeutic benefits it holds a tremendous promise as a natural alternative in the field of oral medicine especially in the management of oral mucosal lesions, oral potentially malignant lesions such as lichen planus and leukoplakia, candidiasis, oral cancer and oral mucositis. Further animal studies and clinical trials are recommended to fully reap the therapeutics potential of raspberry extract.

## Introduction

Plants are vital sources for providing individuals with nutrient-dense sustenance. They produce a variety of secondary metabolites via various chemical pathways, thereby helping the human body combat various kinds of infections, underscoring the significance of plants in our health [1]. Plants contain numerous bioactive compounds which have various therapeutic properties that are essential for human health [2]. Fruits have a high level of polyphenolic compound content, which is beneficial for human health [3]. Fruits contain vital antioxidants, dietary fiber, and nutrients that are necessary for the person’s body to improve digestion, lower blood pressure, decrease heart-related diseases, and improve the health of the skin [4]. *Rubus idaeus *(Family: Rosaceae) commonly called raspberry, is a red edible fruit that is cultivated in Central Asia, Europe, and many other places [5].

Raspberry fruits have been used for therapeutic purposes for a long time, and the compounds present in the fruit may have antioxidant effects and aid in the relaxation of blood vessels [6]. Raspberry fruit supplies potassium, which is necessary for cardiovascular functions; it is shown to decrease blood pressure; and it contains manganese, which is essential for bone health [7]. The bioactive compound present in the raspberry fruit alters lipid metabolism, specifically by enhancing lipolysis within adipocytes, to serve as an anti-obesity drug [8]. The phytochemicals and two main antioxidants, ellagitannins and anthocyanins, present in the raspberry have the ability to prevent the growth of tumors [9]. The leaves of raspberry were used for the treatment of pain relief during menstrual periods, moderate inflammation, and diarrhea [10]. Two antioxidants, namely, lutein and zeaxanthin, found in raspberry fruits are essential in preserving ocular health and they have a significant level of vitamin C, which boosts immunity against many diseases and infections [11,12]. 

Raspberries possess polyphenols that may have the ability to control insulin levels by lowering inflammation and oxidative stress, which are the three major causes of Alzheimer’s disease [13]. In order to alleviate coughing and diarrhea, crushed raspberry roots were employed as an astringent. Raspberry tea was used for reducing labor contractions and for the treatment of throat and stomach problems [14]. Raspberry is used to support the uterus throughout pregnancy, enhance labor outcomes, and reduce excessive postpartum bleeding [15]. The objectives of this invitro study are to assess the antimicrobial activity of *R. idaeus* aqueous extract against *Streptococcus mutans *and *Candida albicans*, while also evaluating the extract's antioxidant capabilities through DPPH ((2, 2-Diphenyl-1-picrylhydrazyl hydrate)) and hydrogen peroxide radical scavenging assays. Additionally, the study seeks to investigate the anti-inflammatory activity using bovine serum albumin (BSA) and egg albumin denaturation assays. By achieving these objectives, the research aimed to provide valuable insights into the therapeutic properties of *R. idaeus*, offering a potential natural and effective alternative for oral healthcare in the treatment of oral potentially malignant lesions, oral cancer, and oral mucositis.

## Materials and methods

Collection and preparation of raspberry fruit extract

**Figure 1:**
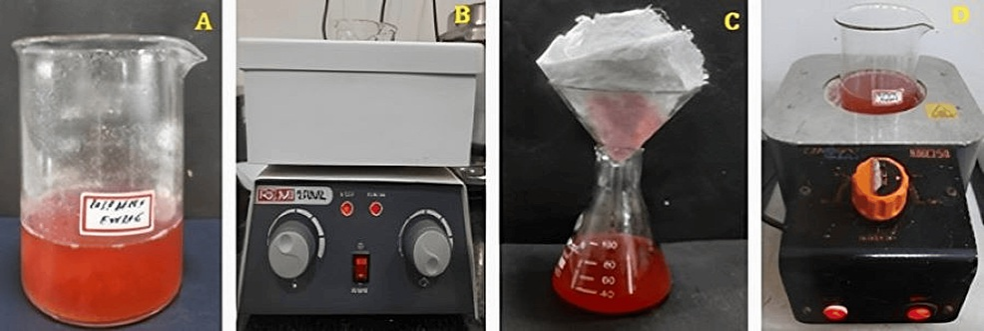
Preparation of raspberry aqueous extract (A) Aqueous solution containing raspberry powder (B) Aqueous raspberry extract heated using heating mantle (C) Raspberry extract filtered using muslin cloth (D) Raspberry extract condensed using heating mantle.

The raspberry fruits were purchased from the local store present in Chennai. The fruits were cleaned with water that had been distilled and then desiccated in an area without sunlight. The dried fruits were finely powdered, and 1 g of fruit powder was added with 100 mL of distilled water as depicted in Figure 1A, and the solution was heated using a heating mantle at 45±5°C for 15 minutes as displayed in Figure 1B. The heated solution was filtered using muslin cloth as shown in Figure 1C, and the filtered solution was then condensed using a heating mantle until it became concentrated to 5 mL as displayed in Figure 1D. The concentrated solution was stored in the refrigerator for further use.

Antimicrobial activity

**Figure 2:**
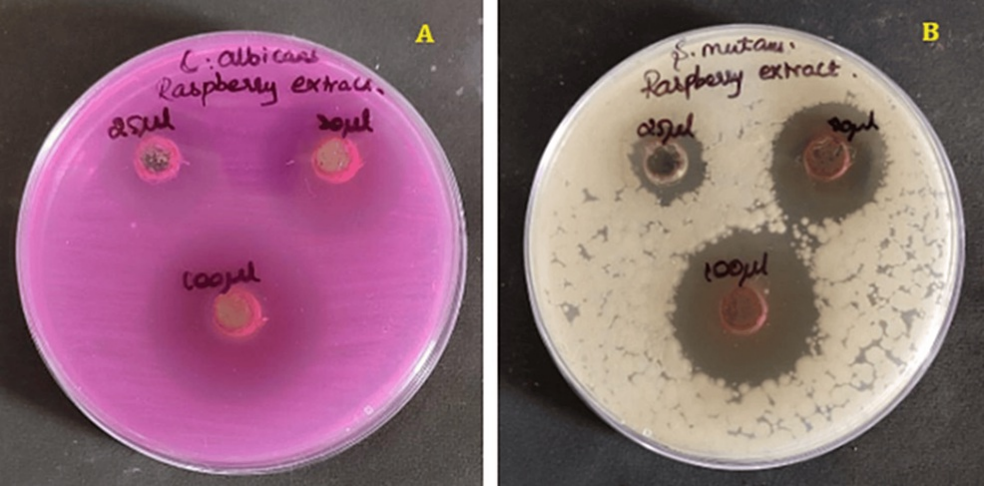
Antimicrobial activity of raspberry extract using agar well diffusion method (A) *Candida albicans* (B) *Streptococcus mutans*

The antimicrobial activity of* Rubus idaeus (R. idaeus)* extract against *Streptococcus mutans (S. mutans) *bacteria and *Candida albicans (C. albicans)* fungi was tested using the agar-well diffusion method. For testing the antimicrobial activity, *C. albicans* fungi were grown in Rose-Bengal agar, and *S. mutans* bacteria were grown in Mueller-Hinton agar. Bacterial and fungal cultures grown in eight-hour-old cultures were used for swabbing the agar plates. For creating wells in the agar plates, an aseptic corn borer with a thickness of 9 mm was used. Then the created wells were filled with the raspberry fruit extracts of varying quantities (25 μL, 50 μL, and 100 μL), and the plates were incubated at room temperature for one day [16]. By estimating the inhibition zone against the target bacteria, the antimicrobial activity of the Raspberry fruit was assessed (Figure 2).

Antioxidant activity

DPPH Assay

The hydrogen-donating ability of the *R. idaeus* fruit extract against DPPH (2, 2-Diphenyl-1-picrylhydrazyl) radicals was scrutinized using a DPPH antioxidant assay. The decolorization of the DPPH present in the methanol solution was used to determine the hydrogen bond-transferring ability of the prepared fruit extract. 1 mL of methanol solution added along with fruit extracts in the concentrations of 10 μg/mL, 20 μg/mL, 30 μg/mL, 40 μg/mL, and 50 μg/mL was further mixed with 2 mL of 1 mM DPPH dissolved in the methanol solution. The prepared solution mixtures were vortexed to mix the solution properly and stored at room temperature in a dark place for 30 minutes. The absorbance of the solution mixtures that were incubated was measured at 517 nm using an ultraviolet-visible spectrophotometer. The methanol solution was used as a blank, and ascorbic acid was employed as a control [17]. The proportion of inhibition of Raspberry fruit extract against DPPH free radicals was estimated using the formula given below:

Percentage of inhibition = Absorbance of control - Absorbance of test sample /Absorbance of control × 100

Hydrogen Peroxide Radical Scavenging Assay

The potential of the *R. idaeus* fruit extract by means of neutralizing hydrogen peroxide radicals was assessed using this assay. A phosphate buffer (50 mM) with pH 7.4 was employed for the preparation of a hydrogen peroxide solution (2 mM). Each tube was filled with 0.1 mL of prepared fruit extract in the concentrations of 10 μg/mL, 20 μg/mL, 30 μg/mL, 40 μg/mL, and 50 μg/mL added with 0.4 mL of phosphate buffer (50 mM) having pH of 7.4, and 0.6 mL of hydrogen peroxide (H2O2) solution. The solution mix was thoroughly vortexed and incubated at room temperature for 10 minutes in a dark environment. Ascorbic acid was used as a standard and for preparing the control solution; the same solution was used without adding any sample or standard [18]. The ability of the prepared fruit extract to scavenge the hydrogen peroxide radicals was calculated using the following formula:

H2O2 scavenging activity = (1 - absorbance of sample/absorbance of control) × 100.

Anti-inflammatory activity

Bovine Serum Albumin Denaturation Assay

The ability of the prepared* R. idaeus* fruit extract to prevent the denaturation of the Bovine Serum Albumin protein (in vitro) was determined using the bovine serum albumin denaturation assay. A solution mixture of 0.5 mL was made using 0.05 mL of the diverse strengths of the prepared fruit extracts (10 μg/mL, 20 μg/mL, 30 μg/mL, 40 μg/mL, and 50 μg/mL) and 0.45 mL of bovine serum albumin. The pH of the developed mixture solution was adjusted to 6.3 using a minimal amount of 1N HCl, and the solution was left to incubate for 3 minutes at 37 °C. All of the tubes were filled with phosphate-buffered saline (2.5 mL) with a pH of 6.3, and 99% dimethyl sulfoxide (DMSO) served as a control. As a means of standard, diclofenac sodium was employed. The entire solution mixture was heated for 10 minutes at 70 °C before cooling for 20 minutes at room temperature. The absorbed wavelength of the solution was determined at 660 nm using a UV-visible spectrophotometer [19]. The percentage of anti-denaturation ability of the prepared fruit extract was measured using the following formula:

Anti-denaturation activity (%) = Absorbance of control - Absorbance of sample/Absorbance of control × 100

Egg albumin denaturation activity

The ability of the prepared *R. idaeus *fruit extract to prevent the denaturation of the egg albumin protein (in vitro) was determined using an egg albumin denaturation assay. A 5 mL solution mixture of egg albumin (0.2 mL), phosphate-buffered saline at pH 6.4, and various amounts of raspberry fruit extract (10μg/mL, 20 μg/mL, 30 μg/mL, 40 μg/mL, and 50 μg/mL) was prepared up to 2 mL. As a control, 99% dimethyl sulfoxide (DMSO) was employed, and diclofenac sodium was used as a standard. The entire mixture was heated for 5 minutes at 70°C before cooling for 15 minutes at room temperature. The absorbance of the solution was measured at 660 nm using a UV-visible spectrophotometer [20]. The percentage of anti-denaturation ability of the prepared fruit extract was measured by means of the following formula:

Anti-denaturation activity (%) = Absorbance of control - Absorbance of sample/Absorbance of control × 100

## Results

**Figure 3:**
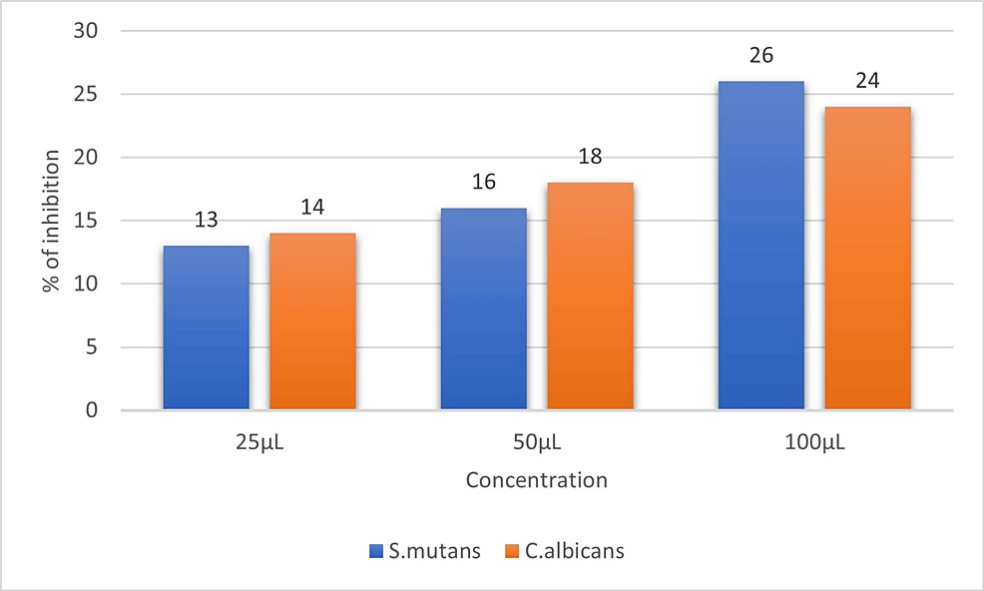
Graph showing the antimicrobial activity of raspberry extract against microorganisms in three varied concentrations of 25, 50, and 100 μL.

The antimicrobial activity of raspberry extract was examined against two pathogens, *S. mutans and C. albicans*, by using the agar-well diffusion method. The prepared extract at 100 µL showed potent antimicrobial activity on *S. mutans *(zone of inhibition-26 mm) and* C. albicans* (zone of inhibition-24 mm). The antimicrobial potential increased as the concentration of the extract was increased (Figure 3).

**Figure 4:**
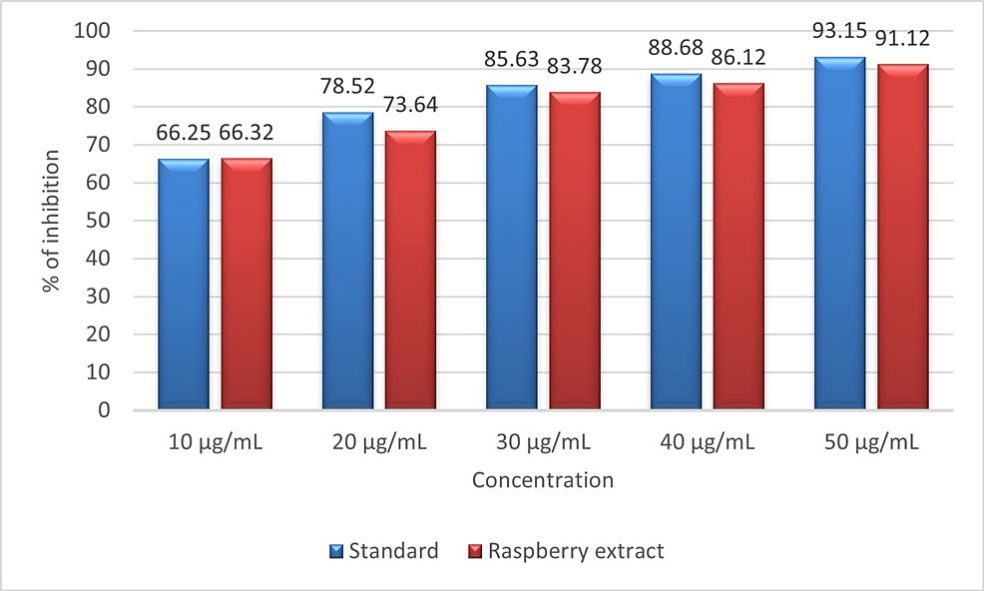
Graph showing the antioxidant activity of raspberry fruit extract against DPPH radicals. DPHH: 2, 2-Diphenyl-1-picrylhydrazyl hydrate

Figure 4 shows the antioxidant activity of the raspberry extract assessed using the DPPH assay. At the highest concentration of 50 µL, the extract shows 91.12% inhibition compared to standard ascorbic acid, which shows 93.15% inhibition against DPPH-stable radicals. At the lowest concentration of 10 µL, the extract shows 64.32% inhibition compared to the standard, which shows 66.25% inhibition against DPPH radicals. The result of the assay shows that the activity of the extract is positively correlated with the increased concentration of the prepared extract against DPPH radicals. 

**Figure 5:**
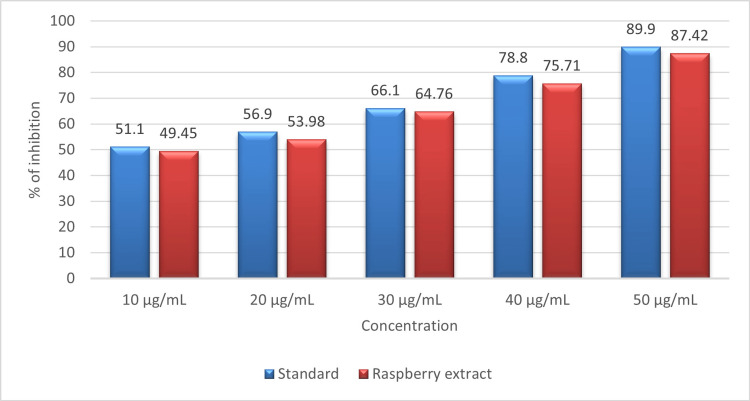
Graph showing free radical scavenging activity of raspberry fruit extract against hydrogen peroxide free radicals.

Figure 5 displays the ability to scavenge free radicals by raspberry fruit extract examined using a hydrogen peroxide assay. At the highest concentration of 50 µL, the extract shows 87.42% inhibition compared to standard ascorbic acid, which shows 89.9% inhibition against hydrogen peroxide free radicals. At the lowest concentration of 10 µL, the extract shows 49.5% inhibition compared to the standard, which shows 51.1% inhibition against hydrogen peroxide free radicals. The result shows that raspberry extract shows potent antioxidant activity against hydrogen peroxide radicals, and the outcome is based on the dose-dependent manner. 

**Figure 6:**
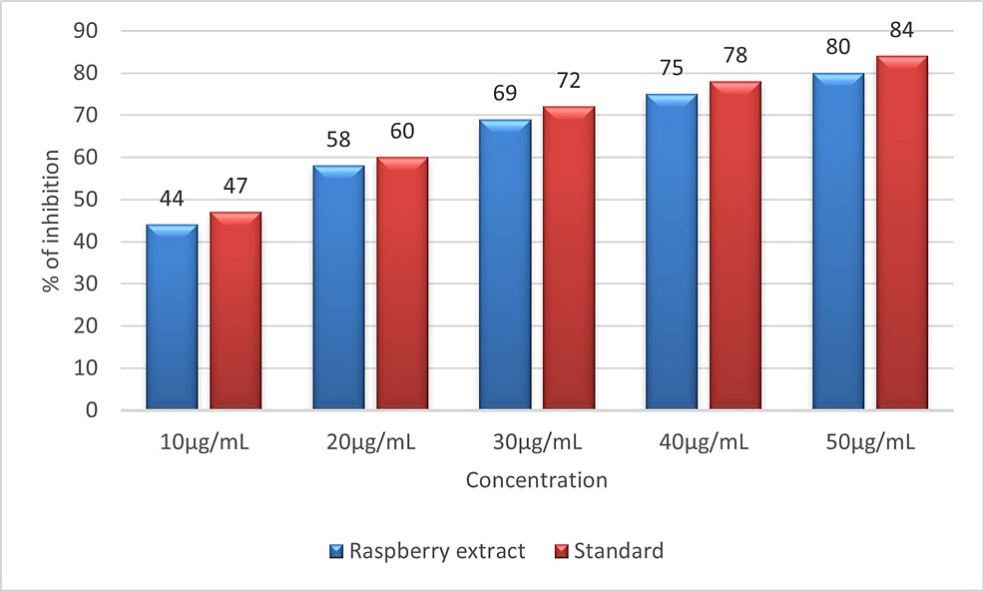
Graph showing the percentage of protein denaturation by raspberry extract utilizing bovine serum albumin denaturation assay.

Figure 6 displays the proportion of inhibition of protein denaturation by raspberry extract on bovine serum albumin protein. At the highest concentration of 50 µL, the extract shows 80% inhibition compared to the standard diclofenac sodium, which shows 84% inhibition on bovine serum albumin protein. At the lowest concentration of 10 µL, the extract shows 44% inhibition compared to the standard diclofenac sodium, which shows 47% inhibition on bovine serum albumin protein. The result of the assay shows that the prepared raspberry aqueous extract has excellent anti-inflammatory activity by preventing the denaturation of bovine serum albumin protein in a dose-dependent manner.

**Figure 7:**
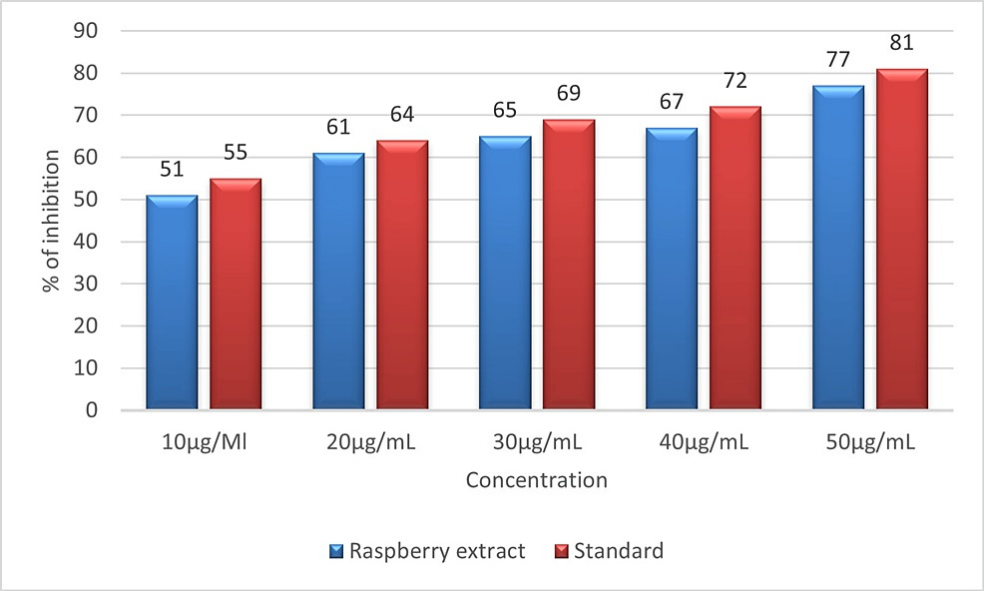
Graph showing the percentage of inhibition of protein denaturation by raspberry extract by means of egg albumin denaturation assay.

Figure 7 shows the proportion of inhibition of protein denaturation by raspberry aqueous extract on egg albumin protein. At the highest concentration of 50 µL, the extract shows 77% inhibition compared to the standard diclofenac sodium, which shows 71% inhibition on egg albumin protein. At the lowest concentration of 10 µL, the extract shows 55% inhibition compared to the standard diclofenac sodium, which shows 51% inhibition. The result reveals that the prepared raspberry extract shows potent anti-inflammatory activity by preventing the denaturation of egg albumin protein in a dose-dependent manner.

## Discussion

Raspberries are widely grown and enjoyed in Asia, Europe, and America and they are linked to blackberries and other brambles or cane berries. Although there are many different sorts of raspberries, the most prevalent are red and black. Raspberry fruits, leaves, and blooms have been used extensively in traditional medical practices [21]. Raspberry fruits contain vitamin C and some B vitamins (thiamin, riboflavin, pantothenic, and folate), and also include significant levels of manganese which are beneficial to health and may lower the risk of chronic diseases in the modern world [11]. Demirbas A et al. (2017) investigated the antibacterial activity of silver nanoparticles using raspberry fruit extract and found its potent antimicrobial activity against *Salmonella enterica subsp. Enterica, Escherichia coli, Staphylococcus aureus, Bacillus cereus, *and *Candida albicans* [22]. Pradeepa et al. 2014 found Raspberry leaf extract with silver nanoparticles exhibited excellent antibacterial activity against *P. aeruginosa, S. aureus, and E. coli *[23]. Krisch et al. (2009) tested raspberry methanol extracts against *Candida albicans *and found a low inhibitory activity (less than 25%) against this pathogen [24]. Nohynek et al. (2006) investigated the antimicrobial properties of 12 Nordic berries against specific human pathogenic microorganisms and noticed phenolic extracts from cloudberry, raspberry, and strawberry, which were all high in ellagitannins, inhibited C. albicans [25]. In the present investigation, the prepared raspberry fruit aqueous extract exhibits strong antibacterial activity in a dose-dependent manner with a subsequent increase in the zone of inhibition against the most common oral opportunistic pathogen, *C. albicans*, and the most common oral disease-causing pathogen, *S. mutans*.

Demibras et al 2017 [22] showed that silver nanoparticles-mediated raspberry fruit extract displayed potent antioxidant activity against DPPH free radicals, whereas Ruttkay-Nedecký found silver nanoparticles-mediated raspberry fruit extract showed moderate activity against DPPH radicals [26]. The highest concentration of 50 µL raspberry extract in the current in vitro assay demonstrated an 87.42% reduction of hydrogen peroxide radicals. Similarly, when compared to standard ascorbic acid, which exhibits 93.15% inhibition against DPPH-stable radicals, the extract exhibits 91.12% inhibition at 50 µL. Moreover, a correlation has been found between higher extract content and increased free radical scavenging activity against hydrogen peroxide and DPPH radicals, confirming the extract's significant antioxidant ability in a dose-dependent manner. Raspberries stand out among other berries due to their remarkably high ellagitannin content and play a noteworthy role accounting for 58% of the fruit's overall antioxidant capacity [27]. Anthocyanin and ellagitannin of red raspberries which are flavonoid chemicals, are what essentially define their polyphenol profile. Anthocyanin accounts for 25% of raspberries' antioxidant capacity and may hinder oxidative enzymes and strengthen the body's defenses against free radical damage [7,21].

In a study done by Chaithanya VM et al, the gel prepared using lycopene and Raspberry plant extract showed significant anti-inflammatory activity on BSA protein [28]. In another study, the nano-herbal formulation prepared using raspberry, lycopene, green tea, and silver nanoparticles showed strong anti-inflammatory activity on BSA protein [29]. There has been no previous study related to the anti-inflammatory activity of Raspberry extract using the egg albumin denaturation assay. Staszowska-Karkut M. et al stated that the primary active constituents responsible for the anti-inflammatory activity of red raspberry leaf are ellagic acid derivatives, followed by catechins [30]. The outcomes of the anti-inflammatory tests in the present study reveal the extract's potential for anti-inflammatory activity, as it outperforms the standard diclofenac sodium at 50 µL concentration, achieving significant inhibition rates of 80% using bovine serum albumin and 77% using egg albumin. Furthermore, there are dose-dependent patterns, highlighting the extract's strong anti-inflammatory properties at higher concentration.

Limitations

As the results are limited to the certain strain of microorganisms under investigation, one of the study's shortcomings is that it may not fully represent the diversity of oral microbes. While the effect of concentration was investigated, it is probable that the therapeutic window was not entirely covered by the concentration range that was chosen. As such, additional studies employing a wider range of concentrations are recommended in order to attain a better understanding of the therapeutic implications.

## Conclusions

*R. idaeus* fruits contain various phytochemical constituents such as polyphenols, flavonoids, and anthocyanins. The bioactive compounds present in these groups have been shown to possess various medicinal properties. Because of its health benefits, raspberry extract is a viable option for proactive oral disease prevention. Additionally, because of its wider anti-inflammatory and antioxidant capabilities, it may have implications for overall systemic health. A natural and complementary approach to maintaining oral health and preventing disease may be made possible by more clinical research and investigation into the uses of raspberry extract in oral medicine, particularly for the management of oral mucosal lesions and oral cancer.
